# Confined molecular catalysts provide an alternative interpretation to the electrochemically reversible demetallation of copper complexes

**DOI:** 10.1038/s41467-022-31661-1

**Published:** 2022-07-22

**Authors:** Etienne Boutin, Aude Salamé, Marc Robert

**Affiliations:** 1 Université Paris Cité, Laboratoire d’Electrochimie Moléculaire, CNRS, F-75006 Paris, France; 2 Institut Universitaire de France (IUF), F-75005 Paris, France

**Keywords:** Catalytic mechanisms, Electrocatalysis, Nanoscale materials

**arising from** Z. Weng et al. *Nature Communications* 10.1038/s41467-018-02819-7 (2018)

Metal phthalocyanines and porphyrins are among the most popular molecular catalysts for the electrochemical reduction of CO_2_. Recently, some copper-based complexes from these ligand families have been reported to promote the formation of methane and even ethylene at a high rate, an unprecedented property for a molecular catalyst^1^. More recently, *operando* X-ray Absorption Spectroscopy studies revealed that under cathodic conditions, small copper nanoparticles were forming from complex demetallation and were responsible for the catalysis^2^. The same studies claimed that the molecular Cu complexes are electrochemically reassembled when the electrode potential goes back to anodic value. Herein, we bring a different interpretation to the latter point, in accordance with all experimental data provided in the original article. Our interpretation accounts for the presence of electrochemically inert metal complexes confined inside the catalytic film and calls for a reassessment of some well-established views.

Recently, Wang et al. investigated the activity of a copper porphyrin (for instance the copper (II)−5,10,15,20-tetrakis(2,6-dihydroxyphenyl)-porphyrin, **PorCu**) as a molecular electrocatalyst deposited onto carbon paper, and they reported the reduction of CO_2_ to CH_4_ and C_2_H_4_ at high current densities in aqueous media (0.5 M KHCO_3_, pH 7.2), an unprecedented result for a molecular catalyst^1^. Although the partition of products was showing a high degree of similarity with the metallic copper electrode, the catalysis was proposed to be molecular, since the spectroscopic signal of the porphyrin was found to be intact after electrolysis. In the following work, the same catalyst along with Cu phthalocyanine (**CuPc**), Cu cyclam, and a copper-based metal-organic framework was investigated with *operando* X-ray Absorption Spectroscopy^2^. The conditions of the previous study were reproduced except that the catalysts were integrated into a Nafion and carbon nanotubes (CNT) matrix, itself deposited on carbon paper. Several potential values were held for at least one hour before an operando XAS analysis was performed. As shown in Figures 2 and S11 of the original article^2^, for both **PorCu** and **CuPc**, Cu nanoparticles (CuNPs) were formed at a potential more negative than −0.65 V vs. RHE. This potential range corresponds to the onset potential for production of CH_4_ and C_2_H_4_, which makes sense considering the known activity of copper particles for CO_2_ electrochemical reduction into CH_4_ and C_2_H_4_^3^, and the frequent observation of metal complex demetallation^4^. Interestingly, the signal of the copper nanoparticles disappears and the XAS signal of the native **PorCu** and **CuPc** reappeared when the electrode potential is set back to its initial value^2^. At first sight, everything happens as if the molecular complexes were demetallated and re-metallated as a function of the applied potential, and this is the conclusion that was taken on by the authors^2^. In the following, we propose an alternative interpretation, consistent with the set of published data^2^, and that we consider more realistic.

First, we question this remetallation proposal since metallating porphyrin or phthalocyanine is usually a slow process that necessitates excess of cation and high temperature to overcome the kinetic barrier, conditions that are not achieved in the original article^2^ (see further discussion in our Supplementary Information). This excludes that CuNPs are anodically dissolved into aqueous Cu^2+^ that would further react with the free base to yield **CuPc** (bottom pathway, Supplementary Fig. 1). Hence, the re-metalation must happen at the CuNPs/Pc^2-^ (particle/ligand) interface, concomitantly with Cu NP oxidation, and before Cu^2+^ goes in the solution (top pathway, Supplementary Fig. 1). In agreement with this scenario, the authors indicated that demetalated phthalocyanine ligands must be in the vicinity of the Cu nanoparticles for the remetallation to take place, and they suggested an appropriated core-shell configuration (Figure 4, original article)^2^. In Supplementary Fig. 2, we represent to scale what such a configuration would represent and show that the possibility for each oxidized Cu to travel back to a complex free site without reaching the solution seems unrealistic, even in a core-shell configuration. The above discussion led us to conclude that the anodic remetallation of **CuPc** is unlikely to occur in any significant proportion. Nevertheless, based on the experiments, the signal of **PorCu** and **CuPc** was recovered at anodic potential, and this requires an alternative explanation.

Our interpretation lies in two important experimental facts. First, the original XAS analysis has been performed in fluorescence mode (Fig. S3, original article)^2^. In such conditions, only the electrode surface area is probed. Notably, if some Cu elements leave the electrode for the electrolyte, their signal will not appear in the spectrum^2^. Second, the XAS analysis is not quantitative since the various spectra are normalized before comparison. For this reason, if the electrode does not have the same loadings of CuPc before and after the experiment, it would still give the same normalized signal, provided there is no other source of Cu element in the film.

At this stage, it is crucial to note that when molecular catalysts are implemented into an ink-based carbon matrix such as the one described here, there is always a significant fraction that will remain electrochemically silent. It has been observed in the case of phthalocyanines^5^ and also quaterpyridines^6^, in films of similar compositions (made from an ink containing a matrix of CNT with Nafion as a binder). In these cases, the concentration of electrochemically addressed catalyst in the film was determined by means of cyclic voltammetry (CV) and was consistently and significantly smaller than the deposited quantity (respectively by 9% and 18%). The reasons for such confinement remain unclear as discussed in the Supplementary Information but its reality is asserted.

Consequently, we propose that only the catalysts that are electrochemically addressed are reduced into CuNPs during the electrolysis and are then oxidized into aqueous Cu^2+^ when the potential is set back to anodic value. As the Cu^2+^ ions leach out into the electrolyte, their signal will fade away, leaving only the normalized signal of the confined and intact catalysts that remained electrochemically inactive all along the process (Fig. 1). The reported data are confirming the presence of such electrochemically confined catalysts inside the film. In the XAS spectra from the original article (Figure 2 and S11)^2^, the characteristic peaks of native **CuPc** or **PorCu** at 8985 eV are still visible, even at the most cathodic potential of −1.06 V vs. RHE. The authors also noticed this fact and performed a fitting of the XAS curves resulting in the estimation that ca. 20% of the original **CuPc** has not been reduced into CuNPs.

**Fig. 1:**
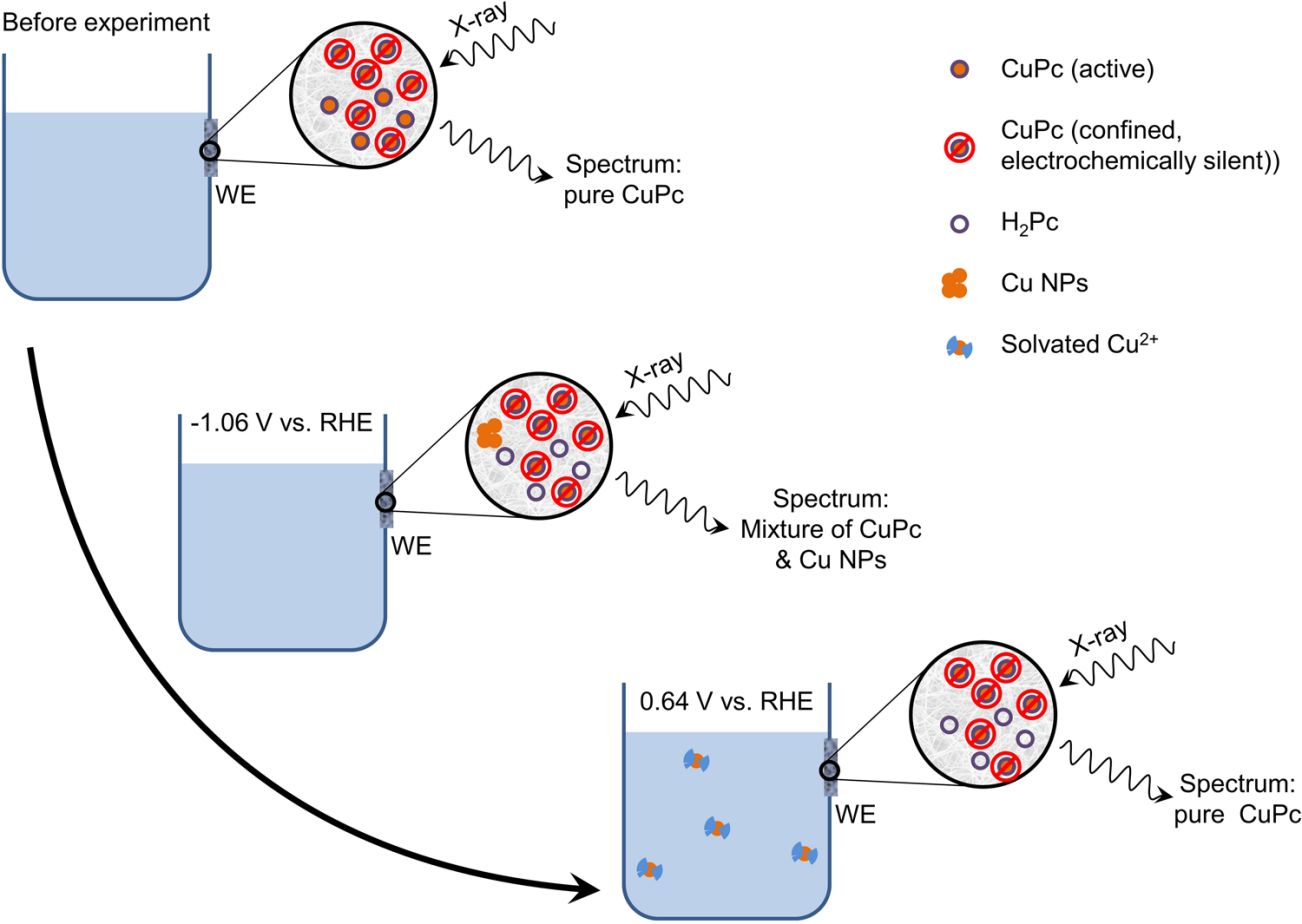
Schematic summary of the alternative interpretation for experimental reported data^2^. In this scenario, the demetallation of **CuPc** is an irreversible process. Likewise, because XAS analysis only probes the Cu element inside the film at the electrode, the normalized spectrum of pure **CuPc** is recovered after the potential is returned to anodic value, while the Cu atoms constituting the CuNPs have leached from the electrode.

Our alternative scenario relies on the possibility for the electrogenerated CuNPs to be fully oxidized into aqueous Cu^2+^ when the potential is set back to +0.64 V vs. RHE. Such fact is consistent with literature data and XRD data provided in the original article as discussed in our Supplementary Information. To definitively confirm such a dissolution process, we prepared an electrode identical to the one reported in the original article^2^. In a cell carefully washed with nitric acid to remove any Cu traces, we reduced **CuPc** in the same conditions (0.5 M KHCO_3_ CO_2_ saturated solution, pH 7.2) at the most negative potential reported (−1.06 V vs. RHE) until the current stabilizes. We then applied the reported anodic potential (+0.64 V vs. RHE) until the current stabilizes and compare the Cu content of the electrolyte before and after the complete procedure through inductively coupled plasma (ICP) analysis. The Cu content quantified in the electrolyte represented between 28 and 41% of the Cu amount initially deposited at the electrode. It is consistent with previous estimations of the catalytically active ratio of phthalocyanine in similar films^5^, and in the same order of magnitude, although slightly below, then the 80% of Cu converted into CuNPs estimated in the original article^2^. All these observations and additional experiments converge to confirm that a significant fraction of the catalyst is either not experiencing any electrochemical process or converted into CuNPs during the cathodic process, followed by Cu^2+^ dissolution during the subsequent anodic process. These two effects explain the reappearance of a pure CuPc signal upon electrode reoxidation. The possibility that the initial interpretation does concomitantly occur to a small extent is not ruled out by the present contribution even if we have shown it is unlikely. Our study sheds light on a parameter that has received little attention so far: the presence of electrochemically inert molecules in catalytic films. It also strongly suggests systematically performing liquid phase analysis after electrolysis to look for ligand fragments or metal ions coming from the catalyst.

## Methods

### Ink preparation

Following the reported procedure^2^, 1.6 mg of MWCNTs and 6.4 mg of CuPc (massic ratio CuPc/MW 4:1) were mixed into 2 mL of methanol. In all, 48 μL of Nafion 5% was added and sonicated for 45 min at 20 °C.

### Film preparation

140 µL of the prepared ink was drop casted on Toray paper (1 cm × 0.5 cm) at room temperature. The electrode was dried in an oven at 100 °C for 30 min. Overall, 0.45 mg of CuPc was deposited on the electrode (0.90 mg/cm^2^).

### Electrochemical reduction of CuPc/MWCNT films

In an electrochemical cell previously washed with nitric acid, 30 mL of a 0.5 M KHCO_3_ solution previously purified (chronopotentiometry at 25 μA cm^−2^ for 18 h) was added and saturated with 1 atm. CO_2_ (pH 7.2).

The electrochemical experiment was performed in a three electrodes configuration with CuPc/MWCNT film as the working electrode, a glassy carbon plate as the counter electrode, and a saturated calomel electrode (SCE) was used as the reference electrode.

An applied negative potential value of −1.73 V vs. SCE (−1.06 V vs. RHE) was set, with iR compensation, until the current stabilizes. The potential was then set back to −0.03 V vs. SCE (0.64 V vs. RHE) until the current stabilize again. The electrolyte after electrolysis was collected and analyzed by ICP so as to quantify the Cu content and it was compared to an electrolyte prior to the electrochemical experiment (see Supplementary information for details and data analysis).

## Supplementary information

Supplementary Information

## Source data

Source Data
