# Supplementary material for: Confined molecular catalysts provide an alternative interpretation to the electrochemically reversible demetallation of copper complexes
Source: Nat Commun. 2022 Jul 22;13:4190. doi: 10.1038/s41467-022-31661-1 (PMC9307776; doi:10.1038/s41467-022-31661-1)
Supplement: Supplementary file 1 — Supplementary Information [file 41467_2022_31661_MOESM1_ESM.pdf]

Suppplementary information

Confined molecular catalyst provide an alternative interpretation to the electrochemically reversible demetalation of copper complexes

Etienne Boutin,[1] Aude Salamé,[1] Marc Robert[1,2]*

[1]Université Paris Cité, Laboratoire d'Electrochimie Moléculaire, CNRS, F-75006 Paris, France.

[2] Institut Universitaire de France (IUF), F-75005 Paris, France.

Calculation of $[Cu^{2+}_{(aq)}]_{max}$ concentration after Cu NPs anodic dissolution.

To obtain the maximum Cu^{2+} concentration, we assume that all the CuPc have been converted into Cu NPs and dissolved back into Cu^{2+} upon application of anodic potential. This maximum concentration is given by the following equation:

$$[Cu^{2+}_{(aq)}]_{max} = \frac{\Gamma_{CuPc,WE} \; S_{WE}}{MW_{CuPc} \; V_{elec.}} = 52 \; \mu M$$

Where:

$\Gamma_{CuPc,WE}$	is the surface mass of CuPc in the working electrode : 1.8 mg cm^{-2}
S_{WE}	is the surface of the working electrode : 0.5 cm^2
MW_{CuPc}	is the molecular weight of CuPc : 576 g mol^{-1}
$V_{elec.}$	is the volume of the electrolyte: 30 mL

Discussion about possible metalation of H_2Pc with aqueous Cu^{2+} after anodic dissolution

The reaction of Cu^{2+} with the free base PorH is actually the last step of **PorCu** synthesis.[1] It typically requires a three-fold excess of Cu^{2+} (6 mM) as compared to PorH (2 mM) and required 19 hours of reflux (T° above 60°C for $CHCl_3$) to achieve a decent (though not complete) metallation yield of 89%. In the conditions where reversible demetallation of **CuPc** and **PorCu** is observed upon XAS, the total amount of Cu initially present on the catalysts would represent a Cu^{2+} concentration of ca. 100 times smaller in the electrolyte after anodic dissolution of the Cu nanoparticles 52 µM for the case of **CuPc**, see above. Also, after demetallation, $Cu^{2+}_{(aq)}$ would be in stoichiometric amount with respect to the ligand, not in excess. In the present case, the environment of the demetallated ligand is unknown as it could either have been transformed into its native protonated form H_2Pc or have been stabilized by potassium cations under the form $2K^+$, Pc^{2-}.

Calculation of $[Cu^{2+}_{(aq)}]_{equ.}$ At 0.64 V vs. RHE

The reaction of interest is:

$$Cu^{2+}_{(aq)} + 2e^- = Cu_{(m)}$$

Its equilibrium is considered with regard to Nernst equation:

$$E = E^\circ - \frac{0.059}{2} \log \frac{1}{[Cu^{2+}_{(aq)}]} \quad \leftrightarrow \quad [Cu^{2+}_{(aq)}]_{equ.} = 10^{\frac{2}{0.059}(E - E^\circ)} = 74 \; \mu M$$

Where:

E = 0.640 V vs. RHE at pH 7.2 = 0.215 V vs. SHE

$E°$ = 0.337 V vs. SHE

Discussion about possible dissolution of CuNP at 0.64V vs. RHE

Our alternative scenario relies on the possibility for the formed Cu NPs to be fully oxidized into aqueous Cu^{2+} at the final applied potential of 0.64 V vs. RHE (0.215 V vs. SHE at pH 7.2).[2] From the standard potential $E°_{Cu^{2+}/Cu}$ = 0.337 V vs SHE[3] and Nernst equation, the equilibrium concentration of Cu^{2+} is calculated to be 74 µM (see above) at this applied anodic potential,

more than the maximum concentration that can be dissolved (52 µM, see above). Even though the total solubility of Cu^{2+} is known to be small in neutral water due to the low solubility products (K_{sp}) of $Cu(OH)_2$ (2.2×10^{-20}),[4] $CuCO_3$ (1.4×10^{-10}),[4] and $Cu_2(OH)_2CO_3$ (1.7×10^{-34}),[5] this only accounts for the solubility of free Cu^{2+}. In the present case, due to HCO_3^- and CO_3^{2-} anionic species present in high concentration, most of the Cu^{2+} will not be in a free form but rather in a complexed form, such as $CuHCO_3^+$, $CuCO_3^0{}_{aq.}$ or $Cu(CO_3)_2^{2-}$. In such buffered solution of carbonate/bicarbonate saturated with CO_2, total dissolved Cu^{2+} concentration can reach more than 10^4 times the theoretical free Cu^{2+} dissolved in solution.[6]

This interpretation is also supported by X-ray diffraction (XRD) measurements made on the samples before and after the experiment. The **CuPc** signal is strongly decreased after electrolysis (Figure 3c from original article). Although XRD is not perfectly quantitative, this important signal diminution matches with a concomitant diminution of the catalyst content at the electrode. The significant dissolution of Cu NPs at 0.64 V vs. RHE is further confirmed by the XRD measurements realized on the other catalysts, namely Cu cyclam and Cu MOF (see Figure 3 of original article) before and after electrolysis. For these compounds that are reduced entirely into Cu NPs, the copper signal (of any form, being Cu, Cu_2O or CuO) is almost imperceptible after electrolysis compared to initial intensity.

It should be noted that in the study under discussion, the recovery of the original catalyst signal does not take place upon simply setting the potential to open-circuit potential (OCP) after the cathodic electrolysis. An anodic potential is subsequently applied for at least one hour before the XAS analysis that features the recovered CuPc signal. Analyzing the electrode directly after electrolysis without anodic post-treatment would have immediately shown the presence of Cu based NPs issued from the catalyst decomposition at the electrode surface.

Discussion about molecule electrochemical inertness in film

The electrochemical inertness of some Cu complexes in the catalytic film deposited at the electrode is a fact that has already been reported. But the reason for such confinement and its proportion remains unclear. It can be that some of the catalysts are drowned in the Nafion polymeric matrix or located inside hydrophobic pockets formed by carbon nanotubes entanglement. In each of these cases, the following reactions would not occur:

$$CuPc + 2e^- + 2\,H^+ \rightarrow H_2Pc + Cu_{(m,\,NP)}$$

$$CuPc + 2e^- + 2\,K^+ \rightarrow 2K^+, Pc^{2-} + Cu_{(m,\,NP)}$$

in the former case, because of the absence of electron since Nafion is an insulator, and in the latter case due to the lack of protons (or potassium cation) as the solvent cannot access the hydrophobic pocket.

Discussion about precedent reversible processes

As stated by the authors of the original article, reversible process had already been reported in chemical and sometimes electrochemical conditions. They concern crystallographic surface organization of solid-state material,[9–15] nanoparticles composition,[16] but not metal complexes.

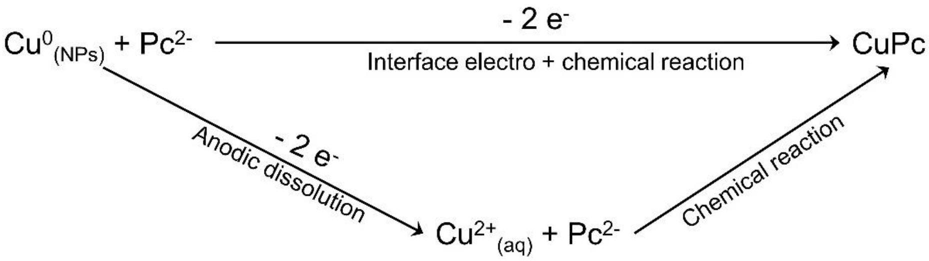

Supplementary Figure 1. Possible pathways for CuNPs anodic reaction with Pc^{2-} to give back CuPc. Top pathway: reaction at the CuNPs/Pc^{2-} interface. Bottow, two steps pathway: pathway involving the anodic dissolution of Cu NPs into aqueous Cu^{2+} followed by its subsequent reaction with Pc^{2-}. In the figure, Pc^{2-} stands for either H_2Pc or K_2Pc which are the two most likely configurations of the demetallated ligand.

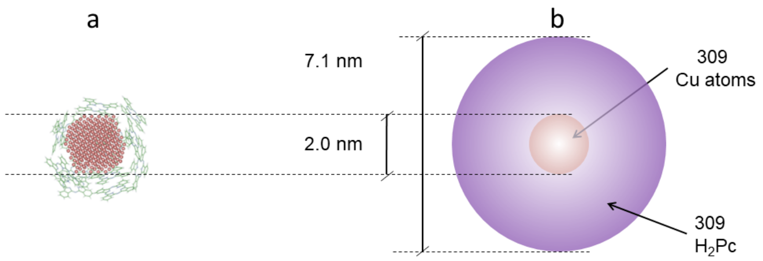

Supplementary Figure 2. Schematic representations of Cu nanoparticles surrounded by phthalocyanine ligands. a) Originally proposed organization of H_2Pc molecules around a CuNP of 309 atoms to enable reversible demetallation, reproduced from original paper.[7] b) To scale representation of a dense matrix of 309 H_2Pc molecules around a 309 atoms Cu NP.

Supplementary Figure 2 represents the same 2 nm diameter nanoparticle composed of 309 atoms[7] (similar to Figure 2a), surrounded by 309 H_2Pc molecules in dense crystallographic form (1.43 g cm^{-3} under β-form).[8] The surrounding H_2Pc layer represents a 7.1 nm diameter equivalent sphere (Figure 2b), implying that most of the ligands are not at the vicinity of the Cu NPs interface as required by the original interpretation. The reasoning also applies to **PorCu** as the ligand is larger and should occupy an even larger volume. Even under the most favorable conditions that we consider here (i.e. CuNPs encapsulated by free ligands, packed organization of ligands) the possibility for each oxidized Cu to travel back to a complex free site without reaching the solution seems unrealistic.

Cu^{2+} quantification by optical ICP after electrochemical reduction of CuPc/MWCNT films.

Following the protocol described in the Methods section of the main text, the electrolyte was collected after electrolysis and analyzed by ICP. An optical-ICP ICAP 6300 ThermoElectron apparatus was used with the following characteristics:

- Wavelengths: 165-847 nm
- RACID diode stripe detector
- Argon plasma: 750 à 1350 W (analysis at 1150 W)
- Peristaltic pump: 0 to 125 rpm (analysis at 50 rpm)
- Detection limit: 1 ppb for most of the elements
- Analysis range: 2 ppb to 1 ppm (axial view)// 1 to 10 ppm (radial view)
- Standard range between 5 and 200 ppb

10 mL of the electrolyte were taken off for ICP analysis. A few drops of HNO_3 were added to the samples before analysis. ICP measurements led to the following results:

**1st experiment**	Cu^{2+} (ppb)	Cu^{2+} (µM)	$Cu^{2+}_{solution}/Cu^{2+}_{deposited}$ molar ratio (cumulated %)
CPE @ -1.06 V/RHE followed by CPE @ - 0.03 V/RHE (2 cycles of 2 h each)	470	7.4	28.5 %

**2nd experiment**	Cu^{2+} (ppb)	Cu^{2+} (µM)	$Cu^{2+}_{solution}/Cu^{2+}_{deposited}$ molar ratio
CPE @ -1.06 V/RHE followed by CPE @ - 0.03 V/RHE (1 cycle)	692	10.9	41.8 %

"Cu^{2+} (ppb)" was directly given by the ICP experiments (raw data). To obtain the molar concentration in Cu^{2+}, the following relationship was used:

$$[Cu^{2+}]_{\mu mol/L} = \frac{[Cu^{2+}]_{ppb}}{M_{Cu^{2+}}}$$

where $M_{Cu^{2+}}$ = 63.546 g/mol.

The $Cu^{2+}_{solution}/Cu^{2+}_{deposited}$ ratio was then calculated knowing the mol number of $Cu^{2+}_{solution}$ and $Cu^{2+}_{deposited}$:

- $Cu^{2+}_{solution}$ is given from $[Cu^{2+}]_{\mu mol/L}$ (the volume of solution in 30 mL).
- $Cu^{2+}_{deposited}$ is given using the total amount of CuPc deposited on the electrode (0.45 mg per 0.5 cm^2, M_{CuPc} = 576 $g.mol^{-1}$).

Supplementary references

1. Weng, Z. *et al.* Electrochemical CO_2 Reduction to Hydrocarbons on a Heterogeneous Molecular Cu Catalyst in Aqueous Solution. *J. Am. Chem. Soc.* **138**, 8076–8079 (2016).
2. Chen, C., Zhang, B., Zhong, J. & Cheng, Z. Selective electrochemical CO2 reduction over highly porous gold films. *J. Mater. Chem. A* **5**, 21955–21964 (2017).
3. Bard, A. J., Parsons, Roger., Jordan, J., & International Union of Pure and Applied Chemistry. *Standard potentials in aqueous solution*. (M. Dekker, 1985).
4. Dean, J. A. & Lange, N. A. *Lange's Handbook of Chemistry*. (McGraw-Hill, 1992).
5. Martell, A. E. & Smith, R. M. *Critical stability constants. 4. Inorganic complexes*. (Plenum Press, 1976).
6. Scaife, J. F. The Solubility of Malachite. *Can. J. Chem.* **35**, 1332–1340 (1957).
7. Weng, Z. *et al.* Active sites of copper-complex catalytic materials for electrochemical carbon dioxide reduction. *Nature Communications* **9**, 415 (2018).
8. Löbbert, G. Phthalocyanines. in *Ullmann's Encyclopedia of Industrial Chemistry, (Ed.)* (2000).
9. Tanaka, K. Chemical reconstruction and catalysis of metal and bimetallic surfaces. *Surface Science* **357–358**, 721–728 (1996).
10. Titmuss, S., Wander, A. & King, D. A. Reconstruction of Clean and Adsorbate-Covered Metal Surfaces. *Chem. Rev.* **96**, 1291–1306 (1996).
11. Kolb, D. M. Reconstruction phenomena at metal-electrolyte interfaces. *Progress in Surface Science* **51**, 109–173 (1996).
12. Eren, B. *et al.* Activation of Cu(111) surface by decomposition into nanoclusters driven by CO adsorption. *Science* **351**, 475 (2016).
13. Karaiskakis, A. N. & Biddinger, E. J. Evaluation of the Impact of Surface Reconstruction on Rough Electrodeposited Copper-Based Catalysts for Carbon Dioxide Electroreduction. *Energy Technology* **5**, 901–910 (2017).
14. Gunathunge, C. M. *et al.* Spectroscopic Observation of Reversible Surface Reconstruction of Copper Electrodes under CO2 Reduction. *J. Phys. Chem. C* **121**, 12337–12344 (2017).

15. Irvine, J. T. S. *et al.* Evolution of the electrochemical interface in high-temperature fuel cells and electrolysers. *Nature Energy* **1**, 15014 (2016).

16. Wei, H. *et al.* In situ Growth of NixCu1-x Alloy Nanocatalysts on Redox-reversible Rutile (Nb,Ti)O4 Towards High-Temperature Carbon Dioxide Electrolysis. *Scientific Reports* **4**, 5156 (2014).
